# Multimodal imaging of a patient with poppers maculopathy

**DOI:** 10.3205/oc000143

**Published:** 2020-04-02

**Authors:** Jale Mentes, Figen Batioglu

**Affiliations:** 1 Ege University, Faculty of Medicine, Department of Ophthalmology, Izmir, Turkey; 2 Ankara University, Faculty of Medicine, Department of Ophthalmology, Ankara, Turkey

**Keywords:** fundus autofluorescence, optical coherence tomography, optical coherence tomography angiography, poppers paculopathy

## Abstract

**Objective:** To describe the findings and the imaging characteristics of a patient with poppers maculopathy.

**Methods:** The ocular findings were evaluated with fundus autofluorescence imaging, spectral optical coherence tomography and optical coherence tomography angiography.

**Results:** A 36-year-old man presented with a history of blurring vision after using poppers. Best-corrected visual acuity was 0.9 and intraocular pressure was normal in both eyes. Fundus examination revealed hyperreflective appearence at macula with a faint foveolar reflex. Optical coherence tomography revealed focal hyperreflectivity and irregularity of the ellipsoid zone at the fovea. Fundus autofluorescence was normal. Optical Coherence Tomography Angiography revealed no vascular changes. The patient received hyperbaric oxygen therapy for 10 days. After one month VA increased, and OCT improved.

**Conclusion:** Inhalation of poppers may be associated with bilateral vision loss due to the disruption of photoreseptors which is clearly demonstrated with OCT.

## Introduction

Poppers have been used for decades in the homosexual communities for their euphoric, aphrodisiac and myorelaxant properties [1], [2]. In central Europe, the possession of the drug is legal but acquirement is prohibited. Their use has been associated with a maculopathy that may cause bilateral vision loss, scotomata, photophobia, and central phosphenes [1], [2], [3]. Bilateral yellow lesions may be seen at the fovea with disruption of outer retinal layers demonstrated with optical coherence tomography (OCT). It is not yet known whether the retinal damage is permanent, although several cases have had long-term reduction in vision in spite of cessation of use [1].

Here, we describe a patient who was seen 15 days after inhaling poppers and who was examined with OCT, optical coherence tomography angiography (OCTA) and fundus autofluorescence (FOF) imaging. 

## Case description

A 36-year-old man presented with a 10–15-day history of blurring vision, unable to focus objects, and central phosphenes. He had a history of using poppers 15 days prior. His medical history included panic attack, mild hypertension and loss of hearing 6 months prior and receiving hyperbaric oxygen therapy.

**Figure 1:**
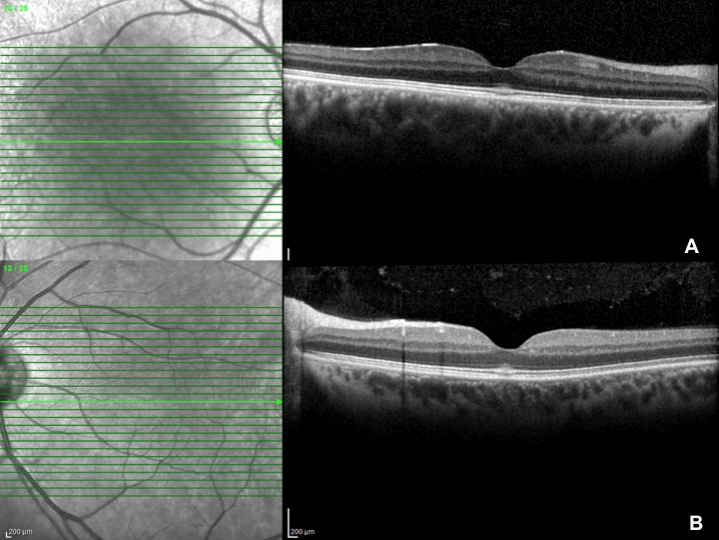
OCT demonstrates hyperreflectivity and irregularity of the ellipsoid zone at the fovea; A) OD, B) OS

**Figure 2:**
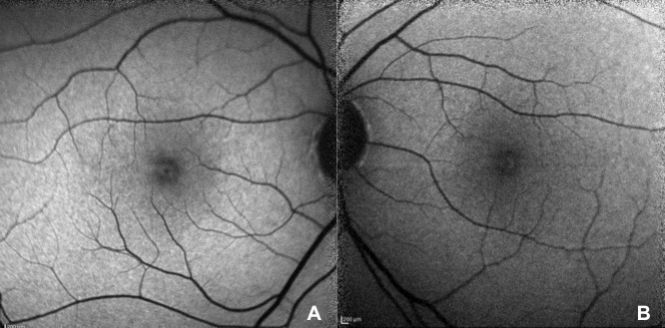
FAF imaging was normal; A) OD, B) OS

**Figure 3:**
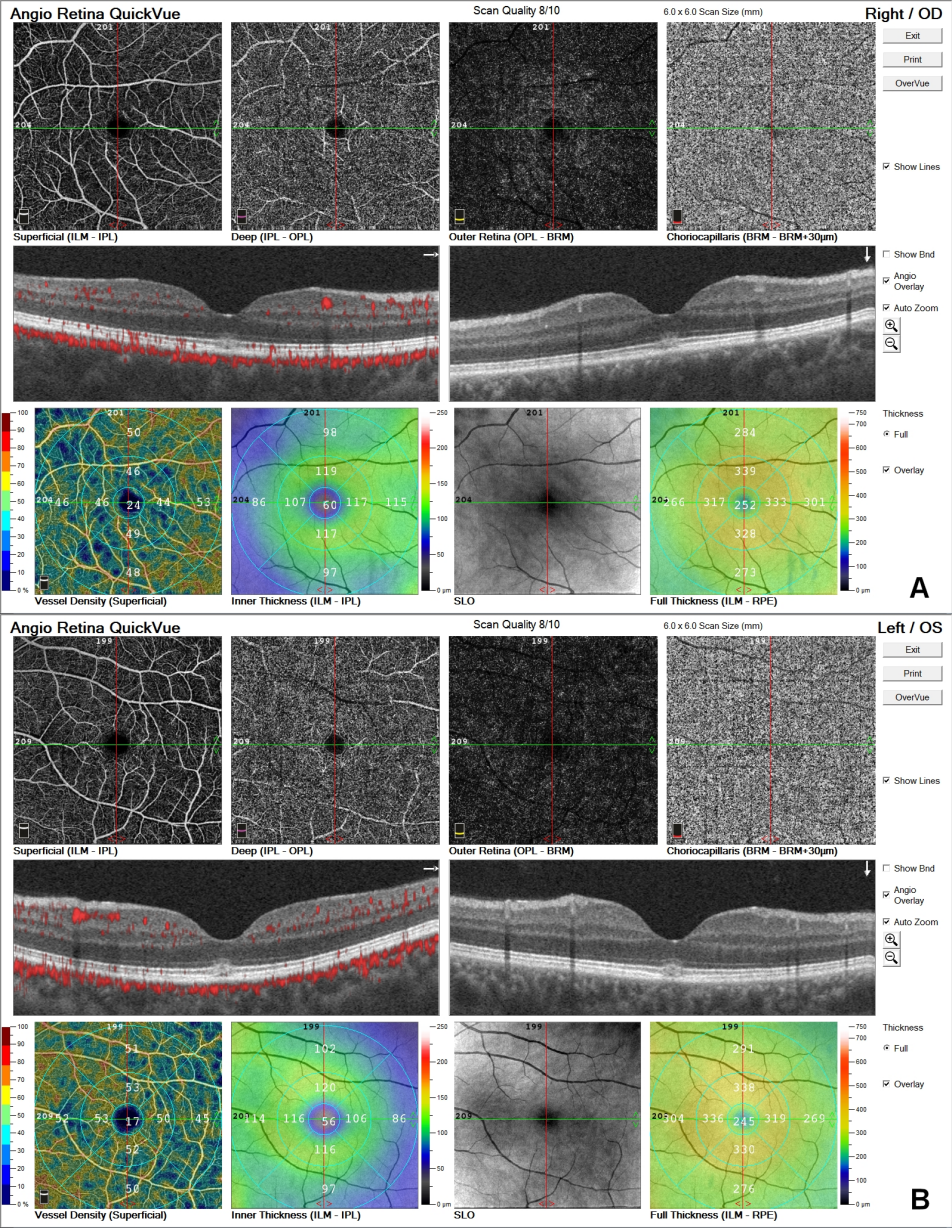
OCTA was normal; A) OD, B) OS

Best-corrected visual acuity was 0.9 and intraocular pressure was 16 mmHg in both eyes. Anterior segment was unremarkable. Fundus examination revealed hyperreflective appearence at macula with a faint foveolar reflex. OCT (Spectralis^®^, Heidelberg Engineering Inc., Heidelberg, Germany) demonstrated focal hyperreflectivity and irregularity of the ellipsoid zone at the fovea (Figure 1A,B (Fig. 1)). No other retinal abnormalities were found. FOF (short wavelength autofluorescence imaging, Heidelberg Spectralis) was normal (Figure 2A,B (Fig. 2)). OCTA (Avanti RT Vue XR^®^ with AngioVue^®^ software; Optovue Inc., Fremont, CA, USA) revealed no vascular changes (Figure 3A,B (Fig. 3)).

The patient was given topical nonsteroidal anti-inflammatory drops, Lutein 10 mg/day (3 months), 1000 mg C vit (10 days), and hyperbaric oxygen (10 days).

**Figure 4:**
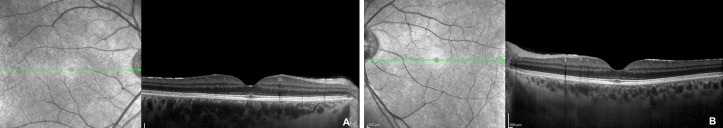
Almost complete resolution of the foveal changes was observed on SD OCT 1 month later; A) OD, B) OS

At 4-week follow-up, the symptoms of the patient decreased and BCVA improved to 1.0 in both eyes. OCT revealed near complete restoration of the ellipsoid zone (Figure 4A,B (Fig. 4)).

**Figure 5:**
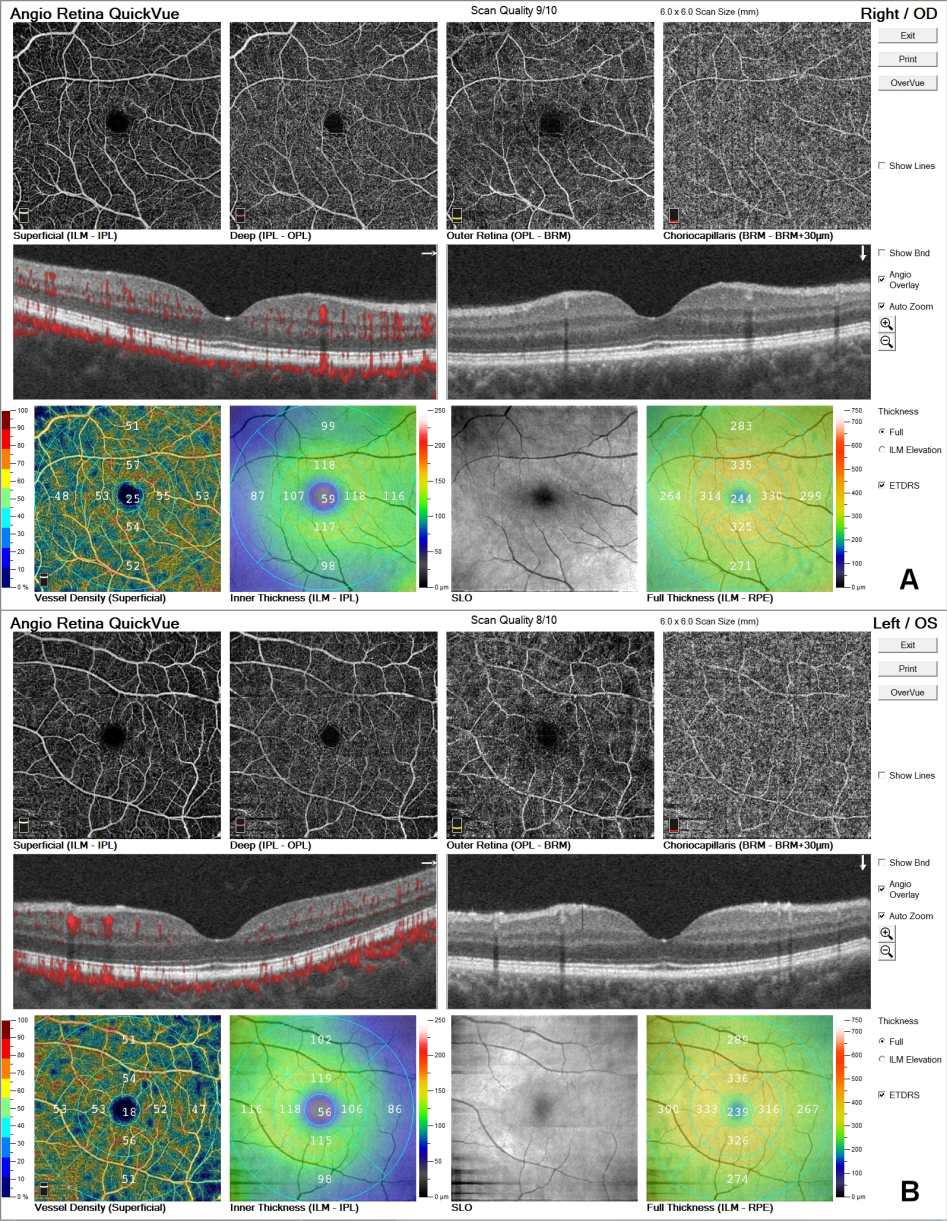
Normal OCTA and OCT at 6^th^ month; A) OD, B) OS

On follow-up examination 3 months later, complaints completely disappeared and fundus examination and OCT were normal. At 6-months visit, OCT and OCTA were unremarkable (Figures 5A,B (Fig. 5)).

## Discussion

Poppers have been used for several decades [4], [5], however poppers maculopathy has been accurately described in recent years [3], [6]. The first report of popper-induced maculopathy was documented by Pece et al. in 2004 [2] in a 30-year-old man. Since then, there has been an increase in the number of reported cases due to a change in the compound from isobutyl nitrite to isopropyl nitrite in 2007, increased usage of the drug, stronger dosages, better detection with spectral domain OCT, and greater awareness by the ophthalmic community [3], [4], [6].

The deleterious effect of alkyl nitrites inhalation on photoreceptors is a well-known fact, but the nature of foveal damage and its relation to visual prognosis remain unclear. Isopropyl nitrite is a very potent nitric oxide donor. Nitric oxide may be directly toxic to the macula, and it has been shown that photoreceptors are among the most sensitive retinal neurons to its toxic effects. No vascular abnormality has been demonstrated so far. Foveal retinal changes were not observed on OCTA in our study as the pathology is based in the avascular zone and is located in the choroid rather than the retina.

Van Bol et al. [7] reviewed 39 patients who presented with outer retinal changes confined to the fovea in the context of poppers use. From the OCT analysis, they observed 3 distinct phenotypes of maculopathy: subfoveal disturbance of the ellipsoid layer, vitelliform-like lesion and microhole. The most common clinical picture was the occurrence of a bilateral yellow central spot corresponding to a subfoveal disturbance of the photoreceptor outer segments on OCT. Our patient had a hyperreflective appearence at macula with a faint foveolar reflex on fundus ophthalmoscopy. OCT revealed focal hyperreflectivity and irregularity of the ellipsoid zone at the fovea as described in the literature.

A detailed history should be taken in these patients because of the similarity of clinical signs with photic maculopathy. In both ‘poppers maculopathy’ and photic maculopathy, there is focal disruption of the IS-OS junction centred at the fovea [1], [4], [8]. Moreover, the size, shape, echogenicity, and temporal evolution of the SD-OCT lesions appear indistinguishable in the two conditions.

Methaemoglobinaemia may develop secondary to the use of ‘poppers’ presenting as altered mental state and unexplained low oxygen saturation [9]. Patients who are unresponsive to standard methylene blue treatment may respond to hyperbaric oxygen therapy [10]. Our patient received 10 days of hyperbaric oxygen in order to treat hypoxia which may cause photoreceptor damage although he did not have methaemoglobinaemia. This was an attempt to support the healing process, not a treatment recommendation, since there is no specific treatment with proven efficacy in this pathology.

## Conclusion

In conclusion, consumers and ophthalmologists should be aware of the possible retinal toxicity of poppers. OCT was often necessary to highlight disruption of central outer segments; on the other hand no vascular abnormality could be identified by OCT angiography.

## Notes

### Competing interests

The authors declare that they have no competing interests.

## References

[1] Davies AJ, Kelly SP, Naylor SG, Bhatt PR, Mathews JP, Sahni J, Haslett R, McKibbin M (2012). Adverse ophthalmic reaction in poppers users: case series of “poppers maculopathy”. Eye (Lond).

[2] Pece A, Patelli F, Milani P, Pierro L (2004). Transient visual loss after amyl Isobutyl nitrite abuse. Semin Ophthalmol.

[3] Vignal-Clermont C, Audo I, Sahel JA, Paques M (2010). Poppers-associated retinal toxicity. N Engl J Med.

[4] Sigell LT, Kapp FT, Fusaro GA, Nelson ED, Falck RS (1978). Popping and snorting volatile nitrites: a current fad for getting high. Am J Psychiatry.

[5] Lockwood B (1996). Poppers: volatile nitrite inhalants. Pharm J.

[6] Audo I, El Sanharawi M, Vignal-Clermont C, Villa A, Morin A, Conrath J, Fompeydie D, Sahel JA, Gocho-Nakashima K, Goureau O, Paques M (2011). Foveal damage in habitual poppers users. Arch Ophthalmol.

[7] Van Bol LB, Kurt RA, Keane PA, Pal B, Sivaprasad S (2017). Clinical Phenotypes of Poppers Maculopathy and Their Links to Visual and Anatomic Recovery. Ophthalmology.

[8] Chen KC, Jung JJ, Aizman A (2012). High definition spectral domain optical coherence tomography findings in three patients with solar retinopathy and review of the literature. Open Ophthalmol J.

[9] McCabe A, McCann B, Kelly P (2012). Pop goes the O2: a case of popper-induced methaemoglobinamia. BMJ Case Rep.

[10] Altintop I, Sanri E, Tatli M, Akcin ME, Denizbasi A (2018). Methemoglobinemia treated with hyperbaric oxygen therapy: A case report. Turk J Emerg Med.

